# Role of Patient Sorting in Avoidable Hospital Stays in Medicare Advantage vs Traditional Medicare

**DOI:** 10.1001/jamahealthforum.2023.3931

**Published:** 2023-11-10

**Authors:** Jianhui (Frank) Xu, Kelly E. Anderson, Angela Liu, Brian J. Miller, Daniel Polsky

**Affiliations:** 1 Department of Health Policy and Management, Bloomberg School of Public Health, Johns Hopkins University, Baltimore, Maryland; 2 Department of Clinical Pharmacy, Skaggs School of Pharmacy and Pharmaceutical Sciences, University of Colorado, Aurora; 3 Division of Hospital Medicine, Department of Medicine, Johns Hopkins University School of Medicine, Baltimore, Maryland; 4 American Enterprise Institute, Washington, DC; 5 Carey Business School, Johns Hopkins University, Baltimore, Maryland

## Abstract

**Importance:**

Unlike traditional Medicare (TM), Medicare Advantage (MA) plans limit in-network care to a specific network of Medicare clinicians. MA plans thus play a role in sorting patients to a subset of clinicians. It is unknown whether the performance of physicians who treat MA and TM beneficiaries is different.

**Objective:**

To examine whether avoidable hospital stay differences between MA and TM can be explained by the primary care clinicians who treat MA and TM beneficiaries.

**Design, Setting, and Participants:**

This was a cross-sectional study of a nationally representative sample of MA and TM beneficiaries in 2019 with any of 5 chronic ambulatory care–sensitive conditions (ACSCs). The relative risk (RR) of avoidable hospital stays in MA compared with TM was estimated with inverse probability of treatment-weighted Poisson regression, both without and with clinician fixed effects. The degree to which the estimated MA vs TM difference could be explained by patient sorting was calculated by comparing the 2 RR estimates. Data were analyzed between February 2022 and April 2023.

**Exposure:**

Enrollment in MA.

**Main Outcome and Measures:**

Whether a beneficiary had avoidable hospital stays in 2019 due to any of the ACSCs. Avoidable hospital stays included both hospitalizations and observation stays.

**Results:**

The study sample comprised 1 323 481 MA beneficiaries (mean [SD] age, 75.4 [7.0] years; 56.9% women; 69.3% White) and 1 965 863 TM beneficiaries (mean [SD] age, 75.9 [7.4] years; 57.1% women; 82.5% White). When controlling for the primary care clinician, the RR of avoidable hospital stays in MA vs TM changed by 2.6 percentage points (95% CI, 1.72-3.50; *P* < .001), suggesting that compared with TM beneficiaries, MA beneficiaries saw clinicians with lower rates of avoidable hospital stays. This effect size was statistically significant to explain the 2% lower rate of avoidable hospital stays in MA than in TM.

**Conclusions and Relevance:**

In this cross-sectional study of MA and TM beneficiaries, the lower rate of avoidable hospital stays among MA beneficiaries than TM beneficiaries was attributable to MA beneficiaries visiting clinicians with lower rates of avoidable hospital stays. The patient sorting that occurs in MA plays a critical role in the lower rates of avoidable hospital stays compared with TM.

## Key Points

**Question:**

Are Medicare Advantage plans’ provider networks associated with beneficiaries’ receiving care from primary care clinicians with lower rates of avoidable hospital stays?

**Findings:**

In this cross-sectional study of 1.32 million Medicare Advantage beneficiaries and 1.97 million traditional Medicare beneficiaries, by controlling for the primary care clinician, relative risk of avoidable hospital stays in Medicare Advantage vs traditional Medicare changed by 2.6 percentage points. This effect size was statistically significant to explain the 2% lower rate of avoidable hospitals stays in Medicare Advantage than in traditional Medicare.

**Meaning:**

The patient sorting that occurs in Medicare Advantage plays a critical role in the lower rates of avoidable hospital stays compared with traditional Medicare.

## Teaser

This cross-sectional study examines whether avoidable hospital stay differences between Medicare Advantage (MA) and traditional Medicare (TM) can be explained by the primary care clinicians who treat MA and TM beneficiaries.

## Introduction

The Medicare program recently passed a milestone, as for the first time, a majority of beneficiaries selected Medicare Advantage (MA) as their preferred form of coverage.^1^ This has brought to the forefront questions regarding the relative effectiveness of the benefit design differences between MA and traditional Medicare (TM), the 2 forms through which beneficiaries access their Medicare benefits. One key difference is that MA plans, unlike TM, construct provider networks (which include physicians, hospitals, and other health care professionals and organizations a plan contracts with) that on average restrict beneficiaries to just over 40% of local physicians for in-network care.^2^ Networks, in conjunction with lower cost sharing for use of in-network clinicians, play a role in sorting patients to specific clinicians.^3^ Thus, in MA, Medicare beneficiaries see a different set of clinicians than if they had TM and could select from virtually all clinicians accepting Medicare.

Hospitalizations due to ambulatory care–sensitive conditions (ACSCs), such as diabetes and hypertension, are potentially avoidable with appropriate primary care and are often used as a measure of primary care quality^4^ and the overall performance of MA relative to TM.^5,6,7^ We test whether avoidable hospitalization differences between MA and TM can be explained by the primary care clinicians seen by MA and TM beneficiaries to examine the role of patient sorting.

## Methods

### Data

We followed the Strengthening the Strengthening the Reporting of Observational Studies in Epidemiology (STROBE) reporting guideline. This study was deemed exempt with the informed consent requirement waived by the Johns Hopkins Bloomberg School of Public Health Institutional Review Board due to the study team’s inability to contact Medicare beneficiaries with deidentified claims. Our primary data sources were the 2018 and 2019 MA encounter data and TM claims data for a nationally representative 20% sample of Medicare beneficiaries. We identified beneficiaries with ACSCs using 2018 claims and assessed their avoidable hospital stays in 2019. We augmented the claims with several data sources. We extracted specialty information of physicians, nurse practitioners, and physician assistants (ie, clinicians) by linking their National Provider Identifier (NPI) with the 2019 IQVIA OneKey health care industry database. We obtained MA plan information and county-level MA penetration from the 2019 public-use Medicare data from the Centers for Medicare & Medicaid Services (CMS). Beneficiary zip code–level socioeconomic information was from the 2016 to 2020 American Community Survey. In addition, we defined local health care markets by grouping counties into commuting zones using a crosswalk by Fowler and Jensen.^8^

### Sample

Full details on sample construction are in eFigure 1 in Supplement 1. We started with beneficiaries continuously enrolled in MA or TM with both Part A and Part B coverage from 2018 to 2019. Among MA beneficiaries, we excluded those who switched plans during either year. Beneficiaries in our sample were also 65 years and older at the beginning of 2018 and resided in the same county in 1 of the 50 states or the District of Columbia through 2018 to 2019.

We further restricted to beneficiaries with 1 or more of 5 chronic ACSCs: diabetes, chronic obstructive pulmonary disease (COPD), asthma, hypertension, and heart failure. The beneficiary-level summary file included Chronic Conditions Data Warehouse (CCW) claim-based chronic condition indicators calculated only using TM claims. Thus, to ensure comparability between MA and TM beneficiaries, we followed the algorithm to derive all conditions indicators for both groups with the 2018 claims.^9^ For the purpose of more balanced MA vs TM comparisons, we excluded diagnoses in chart review records because these records were only available in MA, and prior literature has raised concerns that MA plans might be using medical record review to increase risk scores.^10,11,12^ In a sensitivity analysis, we further excluded diagnoses from health risk assessments, as they are conducted at different rates in MA and TM.^11^

Finally, because our goal was to evaluate the role of patient sorting to clinicians, we narrowed our sample to beneficiaries who we were able to attribute to a primary clinician. For each beneficiary, among all clinicians they saw in 2019, we identified 1 primarily responsible for the beneficiary’s care based on the clinician’s share of the beneficiary’s evaluation and management claims and a specialty hierarchy that assigned descending priority to (1) primary care clinicians, including primary care physicians (PCPs) and nurse practitioners and physician assistants (NPPAs), (2) internal medicine subspecialties, and (3) other physician specialties. For more details about clinician attribution, see eMethods in Supplement 1. After clinician attribution, we excluded clinicians attributed to fewer than 10 Medicare patients in 2019. We tested the effect of selecting this threshold vs 25 in sensitivity analyses.

### Outcome Measures

Our main outcome measure was whether a beneficiary had any avoidable hospital stays in 2019, a binary beneficiary-level measure. We defined avoidable hospital stays to include both hospitalizations and observation stays. This broader definition was used to allow for more balanced MA vs TM comparisons because the rules and incentives governing hospitalizations differ between MA and TM,^7^ and patients in outpatient observation status often receive similar services on the same hospital ward as those in inpatient status. In additional analyses, we separately evaluated the rates of these 2 types of stays in MA and TM.

We identified avoidable hospital stays using the algorithm of the Agency for Healthcare Research and Quality Prevention Quality Indicators, which codes hospital stays tied specifically to an ACSC as potentially avoidable.^4^ For each ACSC, we examined avoidable hospital stays only among beneficiaries with that condition. For analyses that pooled all ACSCs, we examined an overall avoidable hospital stay indicator identifying avoidable stays from any of a beneficiary’s ACSCs.

### Statistical Analysis

We first descriptively compared the rates of avoidable hospital stays in MA and TM for each ACSC and overall. To examine the role of patient sorting in the differences, we focused on the full sample and first estimated the beneficiary-level relative risk (RR) of avoidable hospital stays in MA vs TM. We ran inverse probability of treatment-weighted Poisson regressions to estimate the relative differences between MA and TM and to compare estimates across specifications with different covariates.^13,14^ The key explanatory variable was a binary variable that equaled 1 if the beneficiary enrolled in MA. The main model controlled for beneficiary demographic characteristics (age, sex, race and ethnicity, and dual eligibility for Medicaid in any month), area characteristics (county MA penetration, census region, and zip code–level socioeconomic status, including the high school graduation rate and income levels among older adults), and CCW chronic conditions. Five racial and ethnic groups—Asian/Pacific Islander, Black, Hispanic, White, and other (including American Indian/Alaska Native and other or unspecified races and ethnicities)—were derived from the Research Triangle Institute race variable in the Medicare claims. Using an imputation algorithm, the Research Triangle Institute variable improves the classification of Asian/Pacific Islander individuals and Hispanic individuals in the Medicare enrollment database.^15^ We also included commuting zone fixed effects to account for different geographic concentration of MA and TM beneficiaries. To examine whether our results were sensitive to the inclusion of MA plans submitting a less complete set of records,^16^ we also estimated the model including only MA contracts with a highly complete set of records, as reported in the analysis by Jung and colleagues^17^ based on the concurrence of contracts’ encounter data with Medicare Provider Analysis and Review files and the Healthcare Effectiveness Data and Information Set.

Next, we added clinician fixed effects and compared the estimate with that from the main model. In adding the fixed effects, we estimated the mean difference in avoidable hospital stays between MA and TM within a clinician. From a baseline where avoidable hospital stays were lower in MA, we hypothesized that the RR would increase compared with the main model, indicating a smaller difference between MA and TM among patients of a certain clinician. The change in RR thus captures the degree to which the MA-TM difference can be explained by the sorting of beneficiaries to certain clinicians (eMethods in Supplement 1). This approach relied on clinicians with both MA and TM patients, who accounted for 91% of the beneficiaries in our sample (Table). Note that fixed effects could not be estimated for clinicians with no avoidable hospital stays in their patient population, and they were thus excluded. These clinicians accounted for over 40% of all beneficiaries (Table), and lower-volume clinicians were more likely to be excluded, causing those remaining to mechanically have higher unadjusted rates of avoidable hospital stays among their patients (eFigure 2 in Supplement 1). Therefore, we reestimated the main model with clinicians with 1 or more avoidable hospital stays (the fixed-effects estimation sample) and used the estimate as the baseline to compare estimates with and without clinician fixed effects. We calculated the 95% CI for the difference in the estimates by conducting bootstrap that sampled with replacement at the beneficiary level.

**Table.** Characteristics of Beneficiaries and Primary Clinicians in the Study Sample

Characteristic	Beneficiaries, %		
	MA	TM	Overall
No. of beneficiaries	1 323 481	1 965 863	3 289 344
Age, mean (SD), y	75.4 (7.0)	75.9 (7.4)	75.7 (7.2)
Sex			
Female	56.9	57.1	57.0
Male	43.1	42.9	43.0
Race and ethnicity			
Asian/Pacific Islander	4.4	3.1	3.6
Black	12.1	7.4	9.3
Hispanic	11.9	4.5	7.5
White	69.3	82.5	77.2
Other^a^	2.2	2.6	2.5
Distribution of ACSCs^b^			
Diabetes	39.7	35.8	37.4
COPD	15.5	14.7	15.1
Asthma	6.5	7.0	6.8
Hypertension	90.2	91.7	91.1
Heart failure	14.2	14.2	14.2
Primary clinician characteristics (patient count-weighted)			
Clinician specialty’s patient share^c^			
PCP	87.8	82.9	84.9
NPPA	9.9	14.2	12.5
Internal medicine subspecialties	2.0	2.7	2.4
Other specialties	0.4	0.2	0.2
Had both MA and TM patients	89.1	92.9	91.4
Had MA patients only	10.9	NA	4.4
Had TM patients only	NA	7.1	4.2
Patient count distribution			
10-25 Patients	28.8	30.7	30.0
26-50 Patients	36.8	35.5	36.0
≥51 Patients	34.4	33.7	34.0
Had no avoidable hospital stays among all their patients	46.5	47.2	46.9

Abbreviations: ACSC, ambulatory care–sensitive condition; COPD, chronic obstructive pulmonary disease; MA, Medicare Advantage; NA, not applicable; NPPA, nurse practitioner and physician assistant; PCP, primary care physician; TM, traditional Medicare.

^a^ Other includes American Indian/Alaska Native and other or unspecified races and ethnicities.

^b^ The 5 ACSCs included were diabetes, COPD, asthma, hypertension, and heart failure. They were identified using the 2018 claims following the algorithm of Chronic Conditions Data Warehouse. A beneficiary can have more than 1 ACSC.

^c^ See eTable 1 in Supplement 1 for specialties included in PCP, NPPA, and internal medicine subspecialties.

To demonstrate the intuition behind how clinician fixed effects might change the MA coefficient in the regression above, we show the relative frequency with which MA beneficiaries sorted to high- vs low-performing clinicians (clinicians with low vs high avoidable hospital stays among their patients). The estimated clinician fixed effect—the adjusted rate of avoidable hospital stays by clinician—was used as the performance measure for each clinician. We divided clinicians into deciles, with the first decile including the ones with the highest performance (ie, lowest adjusted rates) and the last including the ones with the lowest performance. We then calculated and plotted each decile’s MA beneficiary share relative to the entire sample’s MA share.

Because MA plans likely steer patients to clinicians based on the plan’s knowledge of clinician past performance, to explore whether our results were robust to past performance and independent of current-year MA and TM patient mix, we used 3 previous years of TM claims (2016-2018) to construct a relatively exogenous avoidable hospitalization performance measure. This measure allowed us to examine the pattern of clinician choice based on an out-of-sample ranking of clinician performance. This alternative definition also allowed for ranking performance for a larger set of clinicians because it was based on 3 years of data rather than 1. We also tested whether results from this analysis and the one above were sensitive to the variation in local MA penetration by dividing the fixed-effects estimation sample into quartiles based on county MA penetration to compare clinician choice among beneficiaries in counties with more similar penetration (eMethods in Supplement 1).

All *P* values were calculated from 2-sided significance tests, and statistical significance was determined using an α = .05. Data analyses were performed between February 2022 and April 2023 using Stata MP, version 17.0 (StataCorp LLC). The main model and the model with clinician fixed effects were estimated with ppmlhdfe,^18^ a user-written command.

## Results

### Characteristics of the Beneficiaries and Primary Clinicians

Our sample comprised 1 323 481 MA beneficiaries (mean [SD] age, 75.4 [7.0] years; 56.9% women; 4.4% Asian/Pacific Islander, 12.1% Black, 11.9% Hispanic, 69.3% White, and 2.2% other race or ethnicity) and 1 965 863 TM beneficiaries (mean [SD] age, 75.9 [7.4] years; 57.1% women; 3.1% Asian/Pacific Islander, 7.4% Black, 4.5% Hispanic, 82.5% White, and 2.6% other race or ethnicity) (Table). Overall, 91.1% had hypertension, followed by diabetes (37.4%), COPD (15.1%), heart failure (14.2%), and asthma (6.8%). Beneficiaries were attributed to 110 594 clinicians, among whom 74% were PCPs and 21% NPPAs, and together PCPs and NPPAs were the primary clinicians for 97% of the beneficiaries. Clinicians who had exclusively MA patients or TM patients were assigned to less than 9% of all beneficiaries. More detailed characteristics of MA and TM beneficiaries included in the analysis are in eTable 3 in Supplement 1. After weighting with the inverse probability of MA enrollment, however, MA and TM beneficiary characteristics and the 2 groups’ propensity of enrolling in MA were balanced (eTable 3 and eFigure 3 in Supplement 1). MA and TM beneficiaries in the fixed-effects estimation sample (ie, beneficiaries attributed to clinicians with 1 or more avoidable hospital stays) were similar to those in the full sample, while there were slight differences in race and ethnicity and census region (eTable 4 in Supplement 1).

### Unadjusted Rates of Avoidable Hospital Stays in MA and TM

**Figure 1.**
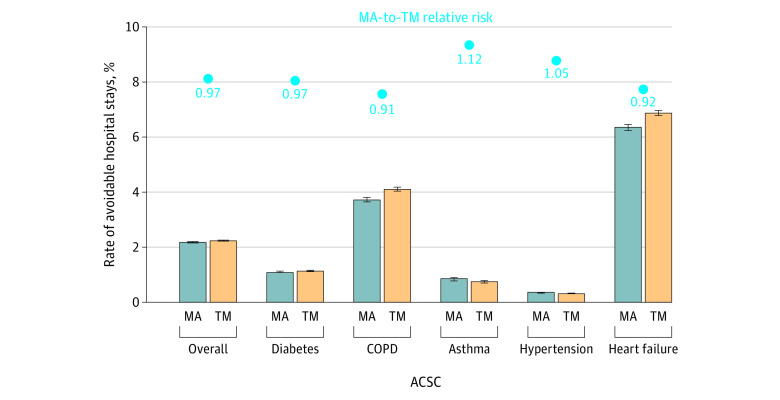
Unadjusted Rates and Medicare Advantage (MA) vs Traditional Medicare (TM) Relative Risk of Avoidable Hospital Stays Overall and by Ambulatory Care–Sensitive Condition (ACSC) Error bars represent 95% CIs. The dots represent unadjusted MA-to-TM relative risk overall and by condition. Avoidable hospital stays included both hospitalizations and observation stays. The 5 ASCSs were identified using 2018 claims following the algorithm of the Chronic Conditions Data Warehouse. COPD indicates chronic obstructive pulmonary disease.

The unadjusted rate of avoidable hospital stays was highest among beneficiaries with heart failure (over 6%) and lowest among those with hypertension (0.3%) (Figure 1). Overall, more than 2% of the beneficiaries had avoidable hospital stays in 2019 due to any of the 5 ACSCs. Unadjusted RRs suggest that MA beneficiaries with diabetes, COPD, and heart failure were 3% to 9% less likely to have avoidable hospital stays than their TM counterparts, but MA beneficiaries with asthma and hypertension were 12% and 5% more likely to have avoidable hospital stays, respectively. The rate of avoidable hospital stays overall was 3% lower in MA.

### Role of Patient Sorting in the Differences Between MA and TM

**Figure 2.**
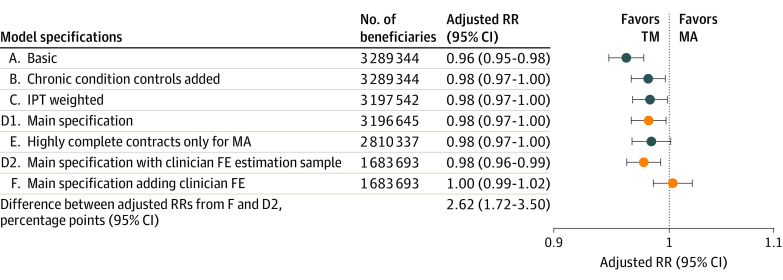
Adjusted Medicare Advantage (MA) vs Traditional Medicare (TM) Relative Risk (RR) of Avoidable Hospital Stays Without and With Clinician Fixed Effects (FE) The basic specification adjusted for demographic and area characteristics only. Chronic conditions and inverse probability of treatment (IPT) weighting were then added sequentially, and the main specification included demographic, area characteristics, chronic conditions, and commuting zone FE and was IPT-weighted. The samples for IPT-weighted regressions included only beneficiaries with a propensity score greater than 0.1 and smaller than 0.9. The fifth specification restricted to contracts with high record completeness only for MA. The clinician FE estimation sample excluded clinicians without any avoidable hospital stays among their patients. Orange dots indicate the main specifications.

Figure 2 presents the overall adjusted RR between MA and TM from various model specifications. The basic specification adjusted for demographic and area characteristics only. Chronic conditions and inverse probability weighting were then added sequentially, and the main specifications are indicated by the orange dots. The fifth specification restricts to MA beneficiaries in contracts with high record completeness and TM beneficiaries. Overall, MA beneficiaries were 1.9% less likely to have avoidable hospital stays than TM beneficiaries (RR, 0.98; 95% CI, 0.97-1.00; *P* = .02), which translates into a likelihood in MA that was 0.04 percentage points lower than in TM. Except for the basic specification (which had the same direction, but a larger effect size), estimates were stable across specifications and remained similar whether all MA contracts or only contracts with high record completeness were included. Avoidable hospitalizations and observation stays demonstrated different patterns (eTable 5 in Supplement 1). The rate of avoidable hospitalizations was 11% lower in MA than in TM (RR, 0.89; 95% CI, 0.88-0.91; *P* < .001). In contrast, while the baseline rates were low, the rate of avoidable observation stays was 66% higher in MA (RR, 1.66; 95% CI, 1.60-1.71; *P* < .001). This is consistent with regulations in MA driving increased use of observation stays over hospitalizations and lends support to our approach of combining hospitalizations and observation stays.

The comparison of the bottom 2 rows in Figure 2 shows the change in the RR of avoidable hospital stays after we included clinician fixed effects. At baseline, the rate of having avoidable hospital stays was 2.3% lower in MA (RR, 0.98; 95% CI, 0.96-0.99; *P* = .003), close to the estimate from the full sample. However, within a certain clinician, MA and TM patients on average had the same rate (RR, 1.00; 95% CI, 0.99-1.02; *P* = .72). The increase in RR from the baseline was 2.6 percentage points and statistically significant (95% CI, 1.72-3.50; *P* < .001). This indicates that the estimated difference between MA and TM was due to MA beneficiaries sorting to different clinicians. Raising the clinician volume threshold to 25 Medicare patients yielded similar results (eFigure 4 in Supplement 1), with a significant difference between RRs with and without fixed effects (1.60 percentage points; 95% CI, 0.60-2.55; *P* < .001). Results were also similar when we further excluded diagnoses in health risk assessments (eFigure 5 in Supplement 1).

**Figure 3.**
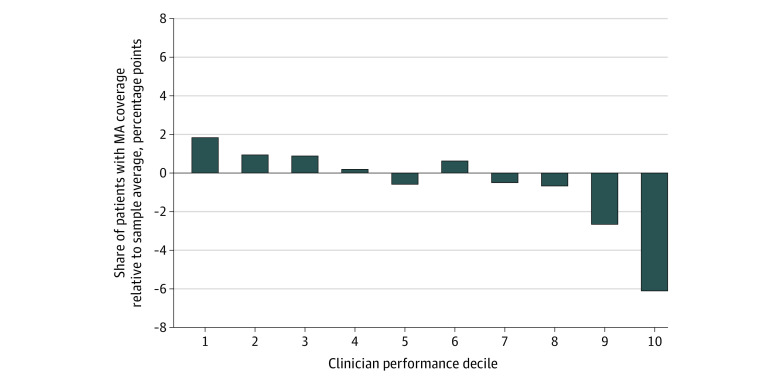
Clinician Performance Decile’s Medicare Advantage (MA) Patient Share Relative to Overall Sample MA Share This figure is based on the clinician fixed-effects estimation sample, which excluded clinicians without any avoidable hospital stays among their patients. The performance deciles were based on estimated clinician fixed effects, with the first decile being the clinicians with the lowest adjusted rates.

Figure 3 illustrates the intuition behind this result, as it shows that MA beneficiaries were more likely than TM to see clinicians with lower rates of avoidable hospital stays and avoid those with higher rates of avoidable hospital stays. In particular, relative to the sample average, the share of MA patients was 1.8 percentage points higher for the top decile. In contrast, the bottom decile received an MA share 6.1 percentage points lower. In eFigure 6 in Supplement 1, we show the relative frequency of MA patient sorting for both the clinician fixed-effects estimation sample and the full sample, using the more exogenous—although out-of-sample—performance measure based on prior years’ TM claims. The above patterns generally persisted in this robustness check; in particular, higher-performing deciles had higher shares of MA patients. Restricting to counties with more similar MA penetration yielded similar results (eFigures 7 and 8 in Supplement 1).

## Discussion

We found that, when controlling for the primary care clinician, the RR of having avoidable hospital stays among MA beneficiaries vs TM beneficiaries changed by 2.6 percentage points, suggesting that MA beneficiaries were more likely than TM beneficiaries to see clinicians with lower rates of avoidable hospital stays and avoid those with higher rates of avoidable hospital stays. This effect size was statistically significant to explain why the observed rate of avoidable hospital stays was 2% lower in MA than TM.

A recent systematic review found that MA was associated with better performance on some measures (more preventive care and fewer emergency department visits and hospitalizations) and uncertain for others, including patient experience, mortality, and disparities in care,^19^ with a subsequent study comparing 2010 and 2017 data demonstrating decreased back surgery and elective hip and knee surgery rates in MA in conjunction with higher quality.^20^

One of multiple mechanisms that contribute to differences in MA performance is the use of provider networks. While networks limit access to a subset of clinicians, prior research suggests that implementing provider networks may add value. Narrow physician networks are associated with higher MA contract star ratings,^2,21^ and MA plans may use networks to promote the prescribing of lower-cost Part B physician-administered drugs when products with comparable clinical effectiveness are available.^22^ Narrower networks in Medicaid and employer-based health insurance reduce spending as enrollees are directed to lower-cost clinicians and obtain fewer necessary and unnecessary services.^23,24^ Our findings suggest that MA networks may serve as an important tool for plans to steer beneficiaries to clinicians with lower rates of avoidable hospital stays.

While this study examines the role of patient sorting in all of MA in relation to TM, we should note that MA plans are not homogeneous. MA plans vary in terms of network breadth, network design (such as preferred provider organizations and health maintenance organizations), and beneficiary cost sharing. Therefore, our results examining avoidable hospital stays and patient sorting reflect the net association of provider networks with MA vs TM. Further research is needed to examine how the role of provider networks varies across MA plans. Relatedly, investigation into the mechanisms through which networks work is warranted, which may include inclusion of high-quality clinicians, inclusion of vertically integrated clinicians, and cost-sharing incentives for in-network care.

However, factors other than MA plans’ steering may also play a role in MA and TM beneficiaries seeing clinicians of different performance. Some beneficiaries may have been visiting the same clinician since before becoming eligible for Medicare and selected an MA plan based on its coverage of their clinician. MA plans can also conduct targeted enrollment outreach to patients of higher-performing clinicians. Future research is necessary, and claims data that allow for observing individuals before and after Medicare eligibility are a promising data source.^25^

### Limitations

This study has several limitations. First, this is an observational study, and the findings are correlational. Second, prior research notes that MA encounter data are incomplete, with increasing completeness over time as policy shifted to basing MA payment on encounter data.^17^ Although our results were similar regardless of whether we restricted to MA contracts with high record completeness, data incompleteness issues may affect the construction of the beneficiary sample and clinician sample and thereby the representativeness of the sample. Third, our clinician fixed effects may be noisy estimates for lower-volume clinicians, and by the nature of fixed-effects analysis, our clinician fixed-effects analysis excluded clinicians with no variation in outcome—clinicians with zero avoidable hospital stays. Thus, our analysis may not generalize to this group of clinicians.

## Conclusions

This cross-sectional study used claims data for both MA and TM beneficiaries to examine whether MA provider networks were associated with beneficiary sorting to primary care clinicians with lower rates of avoidable hospitalizations among their patients. We found that MA beneficiaries’ care was being managed by clinicians with lower rates of avoidable hospital stays. Our findings suggest that provider networks may play an important role in sorting beneficiaries to clinicians with desirable performance metrics.

## Notes

Supplement 1. **eMethods.** Supplemental Methodology **eFigure 1.** Sample Construction Flowchart **eTable 1.** Clinician Specialties **eTable 2.** Clinician Performance Deciles Measured With TM Avoidable Hospitalizations During 2016-2018 **eFigure 2.** Unadjusted Rate of Avoidable Hospital Stays by Clinician Volume in the Full Sample and the Fixed Effect Estimation Sample **eTable 3.** Characteristics of Beneficiaries with Any of the 5 ACSCs **eFigure 3.** Comparing the Distribution of Propensity Scores Among MA and TM Beneficiaries Before and After Inverse Probability Weighting **eTable 4.** Characteristics of Beneficiaries in the Full Sample and in the Fixed Effect Estimation Sample **eTable 5.** Unadjusted rates and Unadjusted and Adjusted MA-to-TM Relative Risk for Avoidable Hospitalizations and Observation Stays **eFigure 4.** Adjusted MA-to-TM Relative Risk of Avoidable Hospital Stays Without and With Clinician Fixed Effects for Beneficiaries With Clinicians With 25 or More Patients **eFigure 5.** Adjusted MA-to-TM Relative Risk of Avoidable Hospital Stays Without and With Clinician Fixed Effects, Excluding Diagnoses in Health Risk Assessments **eFigure 6.** Historical Measure-Based Clinician Performance Decile's MA Patient Share Relative to Overall Sample MA Share **eFigure 7.** Clinician Performance Decile's MA Patient Share Relative to Overall Sample MA Share, by County MA Penetration Quartile **eFigure 8.** Alternative Clinician Performance Decile's MA Patient Share Relative to Overall Sample MA Share, by County MA Penetration Quartile

Supplement 2.
Data Sharing Statement
