# Supplementary material for: Role of Patient Sorting in Avoidable Hospital Stays in Medicare Advantage vs Traditional Medicare
Source: JAMA Health Forum. 2023 Nov 10;4(11):e233931. doi: 10.1001/jamahealthforum.2023.3931 (PMC10638641; doi:10.1001/jamahealthforum.2023.3931)
Supplement: Supplement 1. — eMethods. Supplemental Methodology eFigure 1. Sample Construction Flowchart eTable 1. Clinician Specialties eTable 2. Clinician Performance Deciles Measured With TM Avoidable Hospitalizations During 2016-2018 eFigure 2. Unadjusted Rate of Avoidable Hospital Stays by Clinician Volume in the Full Sample and the Fixed Effect Estimation Sample eTable 3. Characteristics of Beneficiaries with Any of the 5 ACSCs eFigure 3. Comparing the Distribution of Propensity Scores Among MA and TM Beneficiaries Before and After Inverse Probability Weighting eTable 4. Characteristics of Beneficiaries in the Full Sample and in the Fixed Effect Estimation Sample eTable 5. Unadjusted rates and Unadjusted and Adjusted MA-to-TM Relative Risk for Avoidable Hospitalizations and Observation Stays eFigure 4. Adjusted MA-to-TM Relative Risk of Avoidable Hospital Stays Without and With Clinician Fixed Effects for Beneficiaries With Clinicians With 25 or More Patients eFigure 5. Adjusted MA-to-TM Relative Risk of Avoidable Hospital Stays Without and With Clinician Fixed Effects, Excluding Diagnoses in Health Risk Assessments eFigure 6. Historical Measure-Based Clinician Performance Decile's MA Patient Share Relative to Overall Sample MA Share eFigure 7. Clinician Performance Decile's MA Patient Share Relative to Overall Sample MA Share, by County MA Penetration Quartile eFigure 8. Alternative Clinician Performance Decile's MA Patient Share Relative to Overall Sample MA Share, by County MA Penetration Quartile [file jamahealthforum-e233931-s001.pdf]

Supplemental Online Content

Xu JF, Anderson KE, Liu A, Miller BJ, Polsky D. Role of patient sorting in avoidable hospital stays in Medicare Advantage vs traditional Medicare. *JAMA Health Forum.* Published online November 10, 2023. doi:10.1001/jamahealthforum.2023.3931

eMethods. Supplemental Methodology

eFigure 1. Sample Construction Flowchart

eTable 1. Clinician Specialties

eTable 2. Clinician Performance Deciles Measured With TM Avoidable Hospitalizations During 2016-2018

eFigure 2. Unadjusted Rate of Avoidable Hospital Stays by Clinician Volume in the Full Sample and the Fixed Effect Estimation Sample

eTable 3. Characteristics of Beneficiaries with Any of the 5 ACSCs

eFigure 3. Comparing the Distribution of Propensity Scores Among MA and TM Beneficiaries Before and After Inverse Probability Weighting

eTable 4. Characteristics of Beneficiaries in the Full Sample and in the Fixed Effect Estimation Sample

eTable 5. Unadjusted rates and Unadjusted and Adjusted MA-to-TM Relative Risk for Avoidable Hospitalizations and Observation Stays

eFigure 4. Adjusted MA-to-TM Relative Risk of Avoidable Hospital Stays Without and With Clinician Fixed Effects for Beneficiaries With Clinicians With 25 or More Patients

eFigure 5. Adjusted MA-to-TM Relative Risk of Avoidable Hospital Stays Without and With Clinician Fixed Effects, Excluding Diagnoses in Health Risk Assessments

eFigure 6. Historical Measure-Based Clinician Performance Decile's MA Patient Share Relative to Overall Sample MA Share

eFigure 7. Clinician Performance Decile's MA Patient Share Relative to Overall Sample MA Share, by County MA Penetration Quartile

eFigure 8. Alternative Clinician Performance Decile's MA Patient Share Relative to Overall Sample MA Share, by County MA Penetration Quartile

This supplemental material has been provided by the authors to give readers additional information about their work.

© 2023 Xu JF et al. *JAMA Health Forum.*

eMethods. Supplemental Methodology

Clinician Identifier Imputation

One challenge in clinician attribution was the missingness in the claim rendering provider identifier field in MA encounter data. We performed identifier imputation to reduce the missingness among evaluation and management (E&M) claims from 20% to 9%. E&M claims were identified based on Current Procedural Terminology (CPT) codes specified in the Medicare Accountable Care Organization beneficiary assignment algorithm.[1] Despite high missingness in rendering provider NPI in MA encounter data, organization NPI was always populated. We first linked organization NPI to the National Plan and Provider Enumeration System (NPPES) database[2] to obtain entity type information (i.e., whether the NPI represents an individual or an organization) for the NPI. We then took three steps to impute missing rendering NPI:

1. If the organization NPI represents an individual, use it to fill in all missing rendering NPI associated with it. This step reduced missingness from 20.2% to 10.6%.
2. For rendering NPI fields that remain missing, look at the corresponding organization NPI. If the organization NPI is associated with only one nonmissing rendering NPI on E&M claims, use that rendering NPI to fill in all missing rendering NPI fields associated with that organization NPI. This step further reduced missingness to 9.3%.
3. For organization NPIs still associated with missing rendering NPI fields, search if there's one nonmissing rendering NPI that accounts for >=90% of its E&M claims. If so, use that rendering NPI to fill in all missing NPIs associated with the organization NPI. This step further reduced missingness to 9.1%.

Attributing Beneficiaries to a Primary Clinician

We assigned each beneficiary to a primary clinician based on the clinician's share of their E&M claims and a specialty hierarchy that assigned descending priority to the following specialty categories: (1) primary care clinicians, including primary care physicians (PCPs) and nurse practitioners and physician assistants (NPPAs), (2) internal medicine subspecialties, and (3) other physician specialties. The specialties in the first two categories are shown in eTable 1 in Supplement 1. To assign each beneficiary, we first excluded E&M claims for MA beneficiaries where the rendering NPI was still missing. We then took the following steps:

1. If the bene had E&M claims from PCPs or NPPAs, pick the PCP/NPPA with the highest E&M claim share.
2. If there was not a PCP or NPPA, pick the internal medicine subspecialty clinician with the highest E&M claim share.
3. If there was not a PCP, NPPA, or internal medicine subspecialty clinician, pick the clinician with the highest E&M claim share.

Estimating Adjusted Relative Risk of Avoidable Hospital Stays in MA and TM

We estimated relative risk (RR) of having avoidable hospital stays in MA compared with TM with inverse probability of treatment-weighted Poisson regression. We first estimated the

© 2023 Xu JF et al. *JAMA Health Forum.*

propensity score with a logistic model of MA enrollment on demographic characteristics (age, sex, race and ethnicity, and dual-eligibility for Medicaid in any month), area characteristics (county MA penetration, census region, and ZIP-code level socioeconomic status, including fraction of high school graduates and fractions in various income levels among older adults), and CCW chronic conditions. To eliminate extreme weights, we then trimmed the sample to include only beneficiaries with a propensity score greater than 0.1 and smaller than 0.9.[3] Depending on the ACSC, the percentage excluded was between 2% and 3%. The main model we estimated was:

$$\ln(AVOIDABLE\_HOSP\_STAY_i) = \alpha + \beta MA_i + DEMO_i\sigma + AREA_i\gamma + CC_i\mu + \varepsilon_i$$

The key explanatory variable is a binary variable that equals one if the beneficiary *i* enrolled in MA. As the propensity score model, the model includes demographic characteristics, area characteristics, and CCW chronic conditions.

We hypothesized that the rate of avoidable hospital stays was lower in MA than in TM ($RR_1 < 1$).

The Role of Patient Sorting

We added clinician fixed effects to the main model:

$$\ln\left(AVOIDABLE\_HOSP\_STAY_{ip}\right) = \alpha + \beta MA_{ip} + DEMO_{ip}\sigma + AREA_{ip}\gamma + CC_{ip}\mu + \text{CLINICIAN}_p + \varepsilon_{ip}$$

We hypothesized that the resulting $RR_2 > RR_1$, indicating a smaller difference on average between MA and TM among patients of a certain clinician. To evaluate whether the difference in avoidable hospital stays between MA and TM was attributable to MA and TM patients seeing different clinicians, we computed the difference between RR_2 and RR_1 ($RR_2 - RR_1$) and its 95% confidence interval by conducting bootstrap that sampled with replacement at the beneficiary level.

Recovering Clinician Fixed Effects from the Regression

To use them as a performance measure, for each clinician, we extracted the estimate of clinician fixed effect by subtracting their average patient's predicted likelihood of having avoidable hospital stays from the share of their patients that had avoidable hospital stays:

$$\widehat{PROVIDER}_p = \overline{AVOIDABLE\_HOSP\_STAY}_p - \hat{\beta}\overline{MA_p} - \overline{DEMO}_p\hat{\sigma} - \overline{AREA}_p\hat{\gamma} - \overline{CC}_p\hat{\mu}$$

Estimating Clinician Performance with 2016-2018 TM Claims

We estimated a relatively exogenous clinician performance measure—excess avoidable hospitalization—using the 2016-2018 TM claims. For each year, the sample construction was similar to that in the main analysis. The outcome was having avoidable hospitalizations due to any of the 5 ACSCs at the beneficiary level. We stacked three years and estimated a logistic model with covariates including demographic characteristics, area characteristics, CCW chronic conditions, and year fixed effects. Next, we calculated a predicted likelihood of hospitalization

© 2023 Xu JF et al. *JAMA Health Forum.*

for each beneficiary and aggregated it to the clinician level. The performance measure for a clinician was then:

$$\text{Excess avoidable hospitalization} = \text{Share of patients with avoidable hospitalizations} - \text{predicted share}$$

We excluded clinicians with low volume—those with fewer than 30 patients over 2016-2018 or with no patients in 2018. Our performance measure sample in the end included 95,170 clinicians, accounting for 77% of the beneficiaries in the 3-year sample. We divided all clinicians into performance deciles based on excess avoidable hospitalization, with the first decile being the ones with the lowest excess avoidable hospitalization. We show summary statistics of patient count and excess avoidable hospitalization by decile in eTable 2 in Supplement 1.

References

1. Centers for Medicare and Medicaid Services. Medicare Shared Savings Program: Shared Savings and Losses and Assignment Methodology Specifications. Published 2020. https://www.cms.gov/files/document/shared-savings-losses-assignment-spec-v8.pdf-0
2. Centers for Medicare and Medicaid Services. Announcing Changes to the National Plan and Provider Enumeration System (NPPES) Downloadable File. Published 2021. Accessed May 2, 2023. https://www.cms.gov/Regulations-and-Guidance/Administrative-Simplification/NationalProvIdentStand/DataDissemination
3. Crump RK, Hotz VJ, Imbens GW, Mitnik OA. Dealing with limited overlap in estimation of average treatment effects. *Biometrika*. 2009;96(1):187-199. doi:10.1093/biomet/asn055

© 2023 Xu JF et al. *JAMA Health Forum.*

eFigure 1. Sample Construction Flowchart

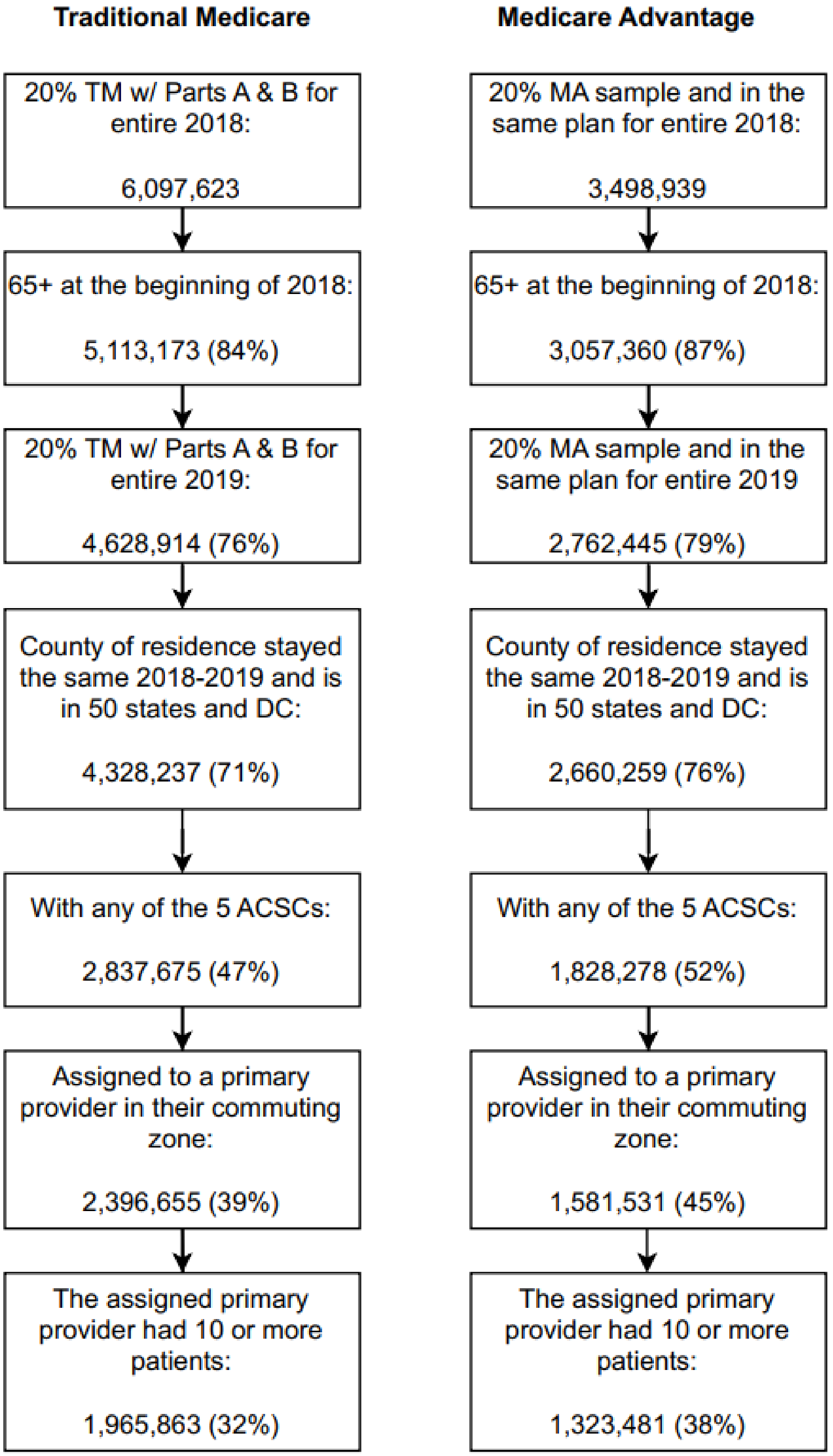

Note: The 5 ambulatory care-sensitive conditions (ASCSs) include diabetes, chronic obstructive pulmonary disease (COPD), asthma, hypertension, and heart failure. They were identified using

© 2023 Xu JF et al. *JAMA Health Forum.*

the 2018 claims following the algorithm of Chronic Conditions Data Warehouse. A beneficiary can have more than one ASCS. MA, Medicare Advantage; TM, traditional Medicare.

© 2023 Xu JF et al. *JAMA Health Forum.*

eTable 1. Clinician Specialties

**PCP**
Family medicine
General practice
Other primary care
Pediatrics
Internal medicine
**NPPA**
Nurse practitioner
Physician assistant
**Internal medicine subspecialties**
Endocrinology
Infectious disease
Nephrology
Rheumatology
Hepatology
Oncology
Pulmonary & critical care medicine
Cardiology
Gastroenterology
Allergy & immunology

Note: PCP, primary care physician; NPPA, nurse practitioner and physician assistant.

© 2023 Xu JF et al. *JAMA Health Forum.*

eTable 2. Clinician Performance Deciles Measured With TM Avoidable Hospitalizations During 2016-2018

	Clinician's total patient count over 2016-2018				Excess avoidable hospitalization (pp)			
Performance decile	Mean	P25	P50	P75	Mean	P25	P50	P75
1	59	37	47	69	-2.8	-3.1	-2.5	-2.2
2	72	40	56	88	-1.7	-1.8	-1.7	-1.6
3	78	41	60	96	-1.3	-1.4	-1.3	-1.2
4	85	43	64	107	-0.9	-1.0	-0.9	-0.9
5	95	45	74	124	-0.6	-0.7	-0.6	-0.6
6	115	62	96	146	-0.2	-0.3	-0.2	-0.1
7	105	56	85	133	0.3	0.2	0.3	0.5
8	91	46	72	117	0.9	0.7	0.9	1.1
9	79	41	63	100	1.8	1.5	1.8	2.1
10	62	38	50	72	4.7	3.1	3.9	5.5

Note: Each decile includes 9,517 clinicians. Each clinician's excess avoidable hospitalization is equal to their actual rate of avoidable hospitalizations less their predicted rate.

© 2023 Xu JF et al. *JAMA Health Forum.*

eFigure 2. Unadjusted Rate of Avoidable Hospital Stays by Clinician Volume in the Full Sample and the Fixed Effect Estimation Sample

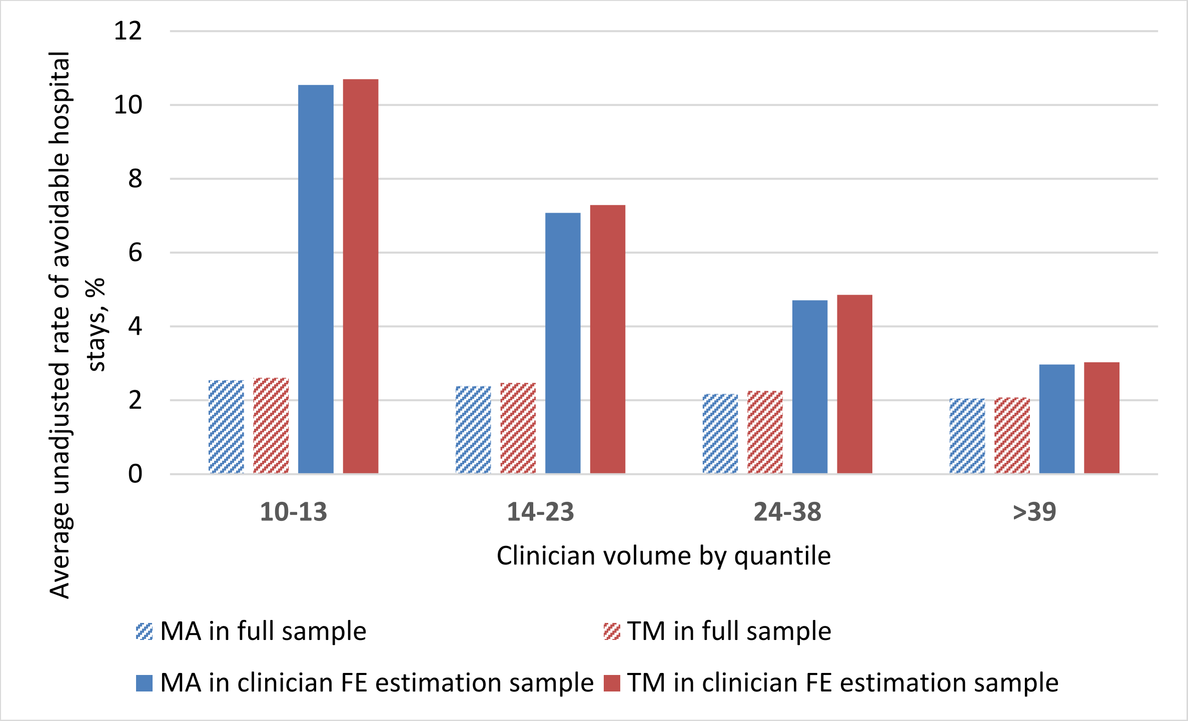

Note: The clinician fixed effect estimation sample excludes clinicians without any avoidable hospital stays among their patients. MA, Medicare Advantage; TM, traditional Medicare.

© 2023 Xu JF et al. *JAMA Health Forum.*

eTable 3. Characteristics of Beneficiaries with Any of the 5 ACSCs

	Unweighted		IPT-weighted	
	MA, %	TM, %	MA, %	TM, %
Mean age	75.4	75.9	75.7	75.7
Female	56.9	57.1	57.0	57.0
Race and ethnicity				
Asian/PI	4.4	3.1	3.7	3.7
Black	12.1	7.4	9.5	9.7
Hispanic	11.9	4.5	7.4	7.5
White	69.3	82.5	77.0	76.8
Other	2.2	2.6	2.4	2.4
Ever dual eligible in year	13.8	11.3	12.7	12.7
County MA penetration	43.3	33.9	38.6	38.5
Census region				
Northeast region	18.1	19.4	19.6	19.5
Midwest region	19.2	21.2	20.8	20.8
South region	39.0	42.2	40.2	40.5
West region	23.7	17.2	19.4	19.3
ZIP code characteristics				
Fraction of high school grads 65+	83.7	86.4	85.3	85.3
Fraction income below FPL among 65+	10.0	8.7	9.2	9.2
Fraction income 100%-400% FPL among 65+	50.7	48.6	49.5	49.6
Fraction income over 400% FPL among 65+	39.4	42.8	41.3	41.2
Chronic conditions				
Mean count of CC	4.7	4.9	4.8	4.8
25th percentile of count	3.0	3.0	3.0	3.0
Median of count	4.0	5.0	4.0	4.0
75th percentile of count	6.0	6.0	6.0	6.0
Alzheimer's Disease	2.2	2.6	2.5	2.5
Alzheimer's Disease and Rltd Disorders or Senile Dementia	6.4	7.8	7.4	7.3
Acute Myocardial Infarction	0.8	0.9	0.9	0.9
Anemia	22.6	27.0	25.4	25.1
Asthma	6.5	7.0	6.8	6.8
Atrial Fibrillation	9.9	12.5	11.4	11.3
Cataract	18.5	22.6	20.7	20.5
Heart Failure	14.2	14.2	14.3	14.2
Chronic Kidney Disease	30.7	26.3	28.1	28.2
Breast Cancer	3.6	4.2	4.0	3.9

© 2023 Xu JF et al. *JAMA Health Forum.*

Colorectal Cancer	1.3	1.4	1.3	1.3
Endometrial Cancer	0.4	0.4	0.4	0.4
Lung Cancer	0.8	1.0	1.0	1.0
Prostate Cancer	4.1	4.6	4.4	4.3
Chronic Obstructive Pulmonary Disease	15.5	14.7	15.0	15.1
Depression	19.5	17.8	18.5	18.5
Diabetes	39.7	35.8	37.5	37.5
Glaucoma	12.3	13.4	12.9	12.8
Hip/Pelvic Fracture	0.6	0.8	0.7	0.7
Hyperlipidemia	70.0	70.8	70.7	70.7
Benign Prostatic Hyperplasia	10.2	10.8	10.6	10.6
Hypertension	90.2	91.7	91.3	91.2
Acquired Hypothyroidism	18.8	21.2	20.3	20.1
Ischemic Heart Disease	28.6	31.9	30.7	30.6
Osteoporosis	8.3	8.8	8.6	8.6
Rheumatoid Arthritis / Osteoarthritis	27.4	30.8	29.4	29.2
Stroke / Transient Ischemic Attack	3.8	4.8	4.4	4.3
N	1,323,481	1,965,863	1,313,813	1,883,729

Note: The 5 ambulatory care-sensitive conditions (ASCSs) include diabetes, chronic obstructive pulmonary disease (COPD), asthma, hypertension, and heart failure. All chronic conditions were identified using the 2018 claims following the algorithm of Chronic Conditions Data Warehouse. Other races/ethnicities include American Indian/Alaska Native and other or unspecified races/ethnicities. MA, Medicare Advantage; TM, traditional Medicare; NH, non-Hispanic; PI, Pacific Islander; IPT, inverse probability of treatment; FPL, federal poverty level; CC, chronic condition.

© 2023 Xu JF et al. *JAMA Health Forum.*

eFigure 3. Comparing the Distribution of Propensity Scores Among MA and TM Beneficiaries Before and After Inverse Probability Weighting

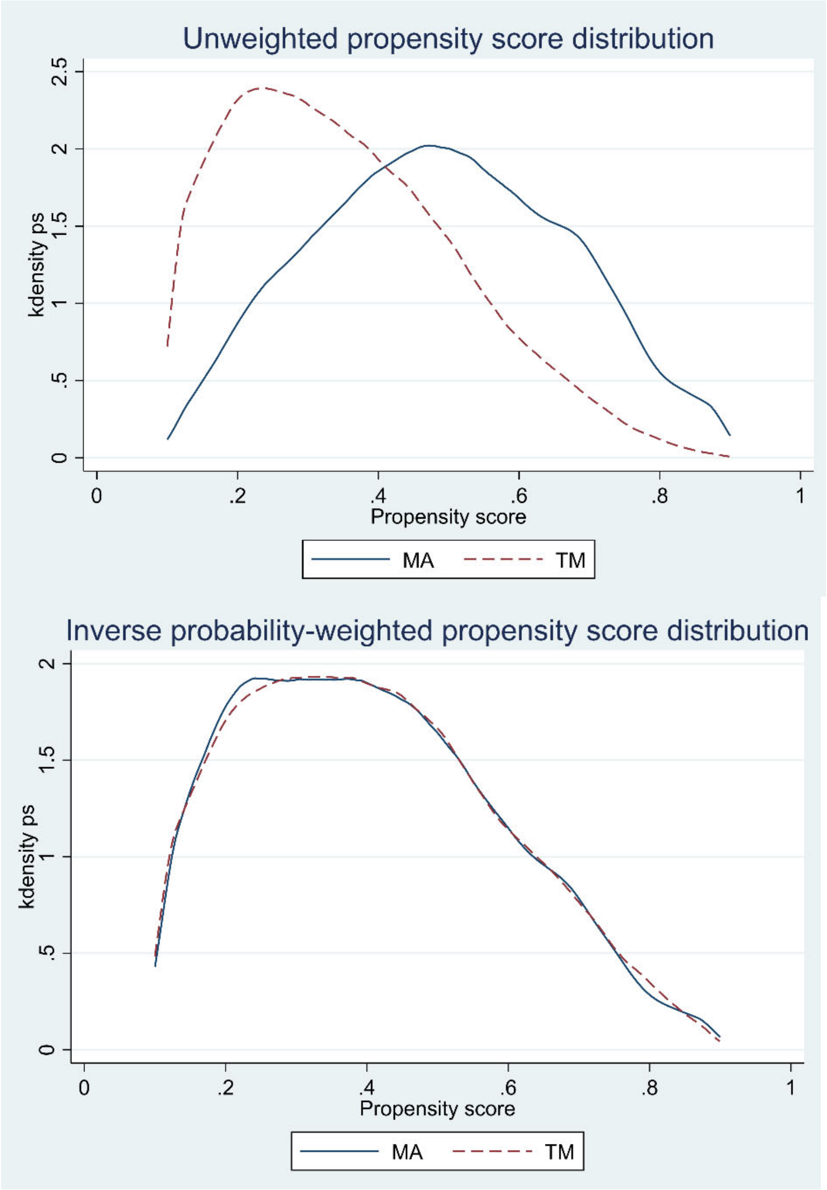

Note: This figure compares the propensity score of MA enrollment. The weighted distribution includes only beneficiaries with a propensity score greater than 0.1 and smaller than 0.9. MA, Medicare Advantage; TM, traditional Medicare.

© 2023 Xu JF et al. *JAMA Health Forum.*

eTable 4. Characteristics of Beneficiaries in the Full Sample and in the Fixed Effect Estimation Sample

	Full sample		Fixed effect estimation sample	
	**MA, %**	**TM, %**	**MA, %**	**TM, %**
Mean age	75.4	75.9	75.6	76.1
Female	56.9	57.1	56.6	56.9
Race and ethnicity				
Asian/PI	4.4	3.1	3.6	2.7
Black	12.1	7.4	13.4	8.3
Hispanic	11.9	4.5	11.9	4.8
White	69.3	82.5	69.0	81.8
Other	2.2	2.6	2.1	2.4
Ever dual eligible in year	13.8	11.3	14.6	12.4
County MA penetration	43.3	33.9	43.3	35.3
Census region				
Northeast region	18.1	19.4	18.0	19.8
Midwest region	19.2	21.2	19.6	21.8
South region	39.0	42.2	43.1	44.2
West region	23.7	17.2	19.3	14.2
ZIP code characteristics				
Fraction of high school grads 65+	83.7	86.4	83.3	85.7
Fraction income below FPL among 65+	10.0	8.7	10.2	9.0
Fraction income 100%-400% FPL among 65+	50.7	48.6	51.6	49.7
Fraction income over 400% FPL among 65+	39.4	42.8	38.2	41.3
Chronic conditions				
Mean count of CC	4.7	4.9	4.8	5.0
25th percentile of count	3.0	3.0	3.0	3.0
Median of count	4.0	5.0	4.0	5.0
75th percentile of count	6.0	6.0	6.0	6.0
Alzheimer's Disease	2.2	2.6	2.4	2.9
Alzheimer's Disease and Rltd Disorders or Senile Dementia	6.4	7.8	7.0	8.7
Acute Myocardial Infarction	0.8	0.9	0.9	1.0
Anemia	22.6	27.0	24.2	28.3
Asthma	6.5	7.0	6.5	7.1
Atrial Fibrillation	9.9	12.5	10.4	13.0
Cataract	18.5	22.6	18.4	21.9
Heart Failure	14.2	14.2	15.8	16.0

© 2023 Xu JF et al. *JAMA Health Forum.*

Chronic Kidney Disease	30.7	26.3	32.3	28.1
Breast Cancer	3.6	4.2	3.6	4.2
Colorectal Cancer	1.3	1.4	1.3	1.4
Endometrial Cancer	0.4	0.4	0.4	0.4
Lung Cancer	0.8	1.0	0.9	1.1
Prostate Cancer	4.1	4.6	4.2	4.6
Chronic Obstructive Pulmonary Disease	15.5	14.7	16.9	16.3
Depression	19.5	17.8	20.0	18.6
Diabetes	39.7	35.8	40.5	37.0
Glaucoma	12.3	13.4	12.5	13.2
Hip/Pelvic Fracture	0.6	0.8	0.6	0.8
Hyperlipidemia	70.0	70.8	71.4	71.9
Benign Prostatic Hyperplasia	10.2	10.8	10.7	11.2
Hypertension	90.2	91.7	90.9	92.2
Acquired Hypothyroidism	18.8	21.2	19.3	21.6
Ischemic Heart Disease	28.6	31.9	30.2	33.3
Osteoporosis	8.3	8.8	8.3	8.9
Rheumatoid Arthritis / Osteoarthritis	27.4	30.8	28.4	31.6
Stroke / Transient Ischemic Attack	3.8	4.8	4.1	5.0
N	1,323,481	1,965,863	700,525	983,168

Note: All chronic conditions were identified using the 2018 claims following the algorithm of Chronic Conditions Data Warehouse. Other races/ethnicities include American Indian/Alaska Native and other or unspecified races/ethnicities. MA, Medicare Advantage; TM, traditional Medicare; PI, Pacific Islander; FPL, federal poverty level; CC, chronic condition.

© 2023 Xu JF et al. *JAMA Health Forum.*

eTable 5. Unadjusted rates and Unadjusted and Adjusted MA-to-TM Relative Risk for Avoidable Hospitalizations and Observation Stays

	Avoidable hospitalizations	Avoidable observation stays
MA rate	1.7%	0.7%
TM rate	1.9%	0.4%
Unadjusted MA-to-TM relative risk	0.87	1.71
Adjusted MA-to-TM relative risk	0.891***	1.657***
	[0.875,0.907]	[1.604,1.712]
N	3,196,460	3,181,110

Note: * *p*<.05, ** *p*<.01, *** *p*<.001. 95% confidence intervals are in brackets. MA, Medicare Advantage; TM, traditional Medicare.

© 2023 Xu JF et al. *JAMA Health Forum.*

eFigure 4. Adjusted MA-to-TM Relative Risk of Avoidable Hospital Stays Without and With Clinician Fixed Effects for Beneficiaries With Clinicians With 25 or More Patients

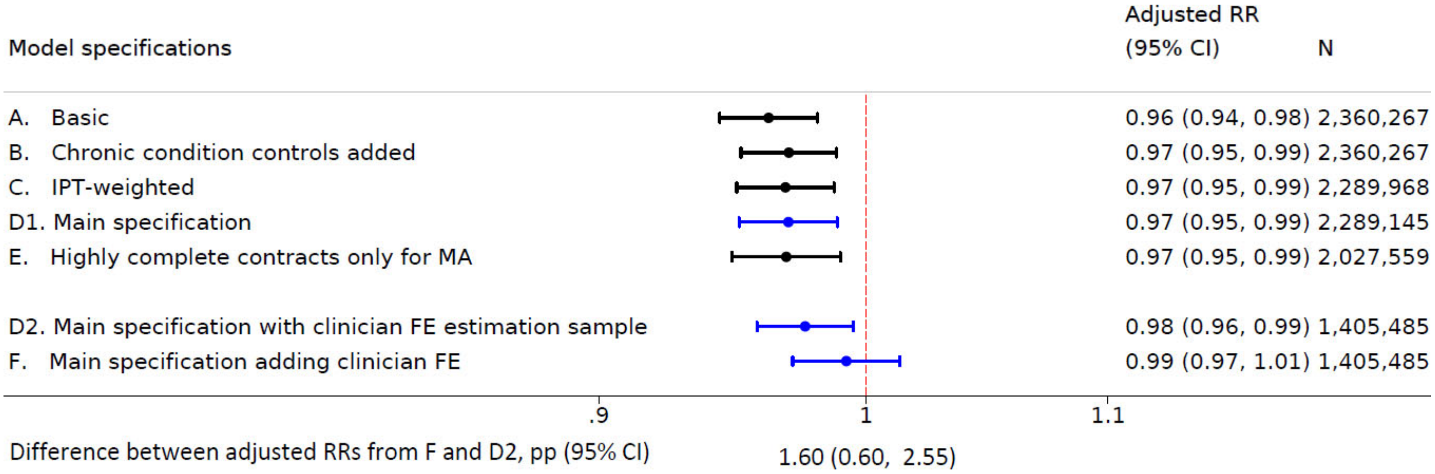

Note: The sample excludes clinicians with fewer than 25 patients. The basic specification adjusts for demographic and area characteristics only. Chronic conditions and inverse probability of treatment (IPT) weighting are then added sequentially, and the main specification includes demographic, area characteristics, chronic conditions, and commuting zone fixed effects and is IPT-weighted. The samples for IPT-weighted regressions include only beneficiaries with a propensity score greater than 0.1 and smaller than 0.9. The fifth specification restricts to contracts with high record completeness only for Medicare Advantage (MA).The clinician fixed effect (FE) estimation sample excludes clinicians without any avoidable hospital stays among their patients. TM, traditional Medicare.

© 2023 Xu JF et al. *JAMA Health Forum.*

eFigure 5. Adjusted MA-to-TM Relative Risk of Avoidable Hospital Stays Without and With Clinician Fixed Effects, Excluding Diagnoses in Health Risk Assessments

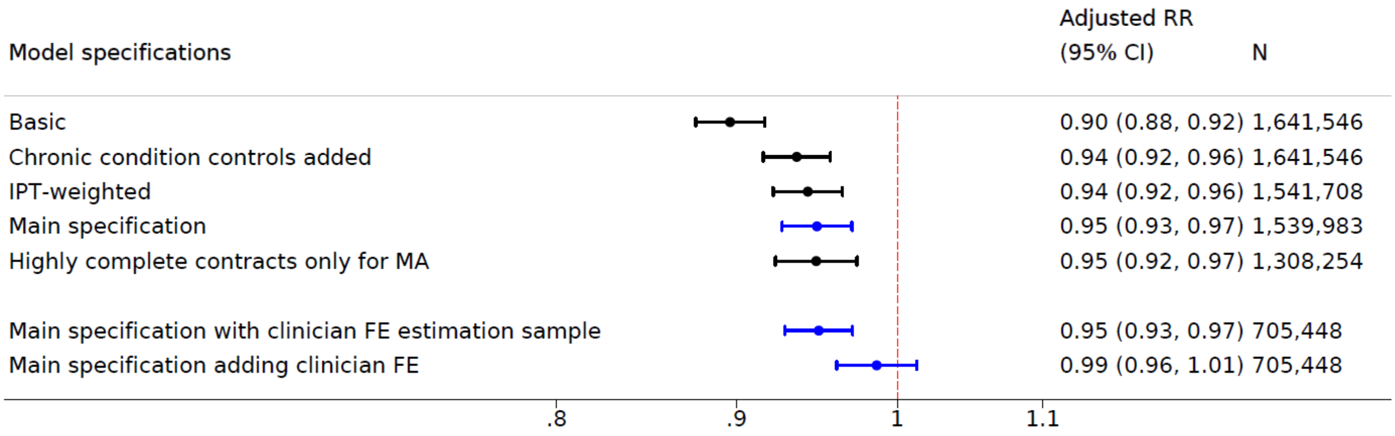

Note: This analysis further excluded diagnoses recorded in health risk assessments in both MA and TM. The basic specification adjusts for demographic and area characteristics only. Chronic conditions and inverse probability of treatment (IPT) weighting are then added sequentially, and the main specification includes demographic, area characteristics, chronic conditions, and commuting zone fixed effects and is IPT-weighted. The samples for IPT-weighted regressions include only beneficiaries with a propensity score greater than 0.1 and smaller than 0.9. The fifth specification restricts to contracts with high record completeness only for Medicare Advantage (MA).The clinician fixed effect (FE) estimation sample excludes clinicians without any avoidable hospital stays among their patients. TM, traditional Medicare.

© 2023 Xu JF et al. *JAMA Health Forum.*

eFigure 6. Historical Measure-Based Clinician Performance Decile's MA Patient Share Relative to Overall Sample MA Share

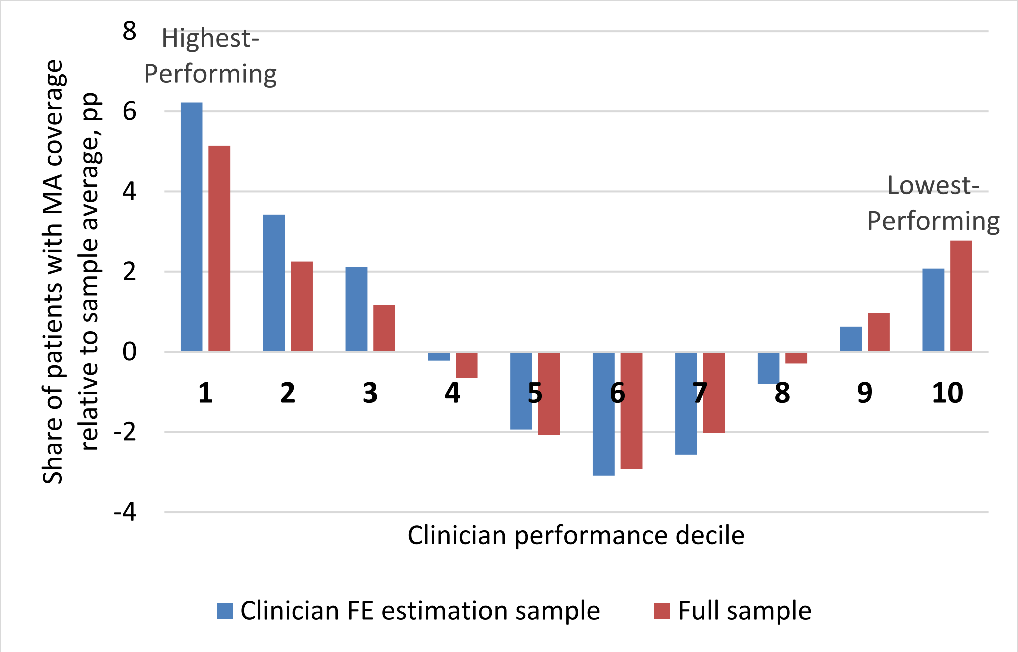

Note: The clinician fixed effect estimation sample excludes clinicians without any avoidable hospital stays among their patients. The performance deciles are based on avoidable hospitalizations among the clinicians' traditional Medicare patients during 2016-2018, with the first decile being the clinicians with the lowest adjusted rates. MA, Medicare Advantage.

© 2023 Xu JF et al. *JAMA Health Forum.*

eFigure 7. Clinician Performance Decile's MA Patient Share Relative to Overall Sample MA Share, by County MA Penetration Quartile

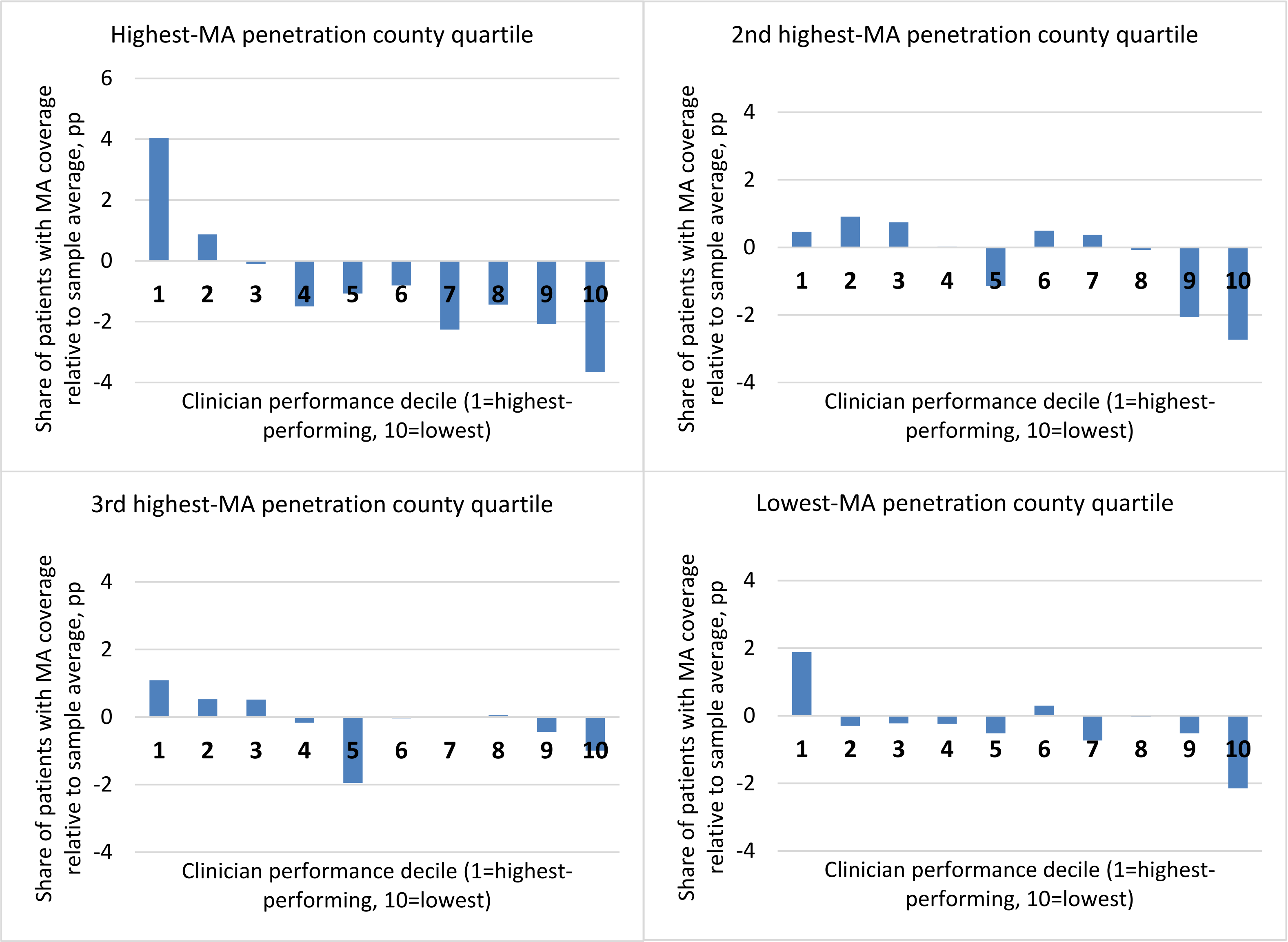

Note: This figure is based on the clinician fixed effect estimation sample, which excludes clinicians without any avoidable hospital stays among their patients. The performance deciles are based on estimated clinician fixed effects, with the first decile being the clinicians with the lowest adjusted rates. MA, Medicare Advantage.

© 2023 Xu JF et al. *JAMA Health Forum.*

eFigure 8. Alternative Clinician Performance Decile's MA Patient Share Relative to Overall Sample MA Share, by County MA Penetration Quartile

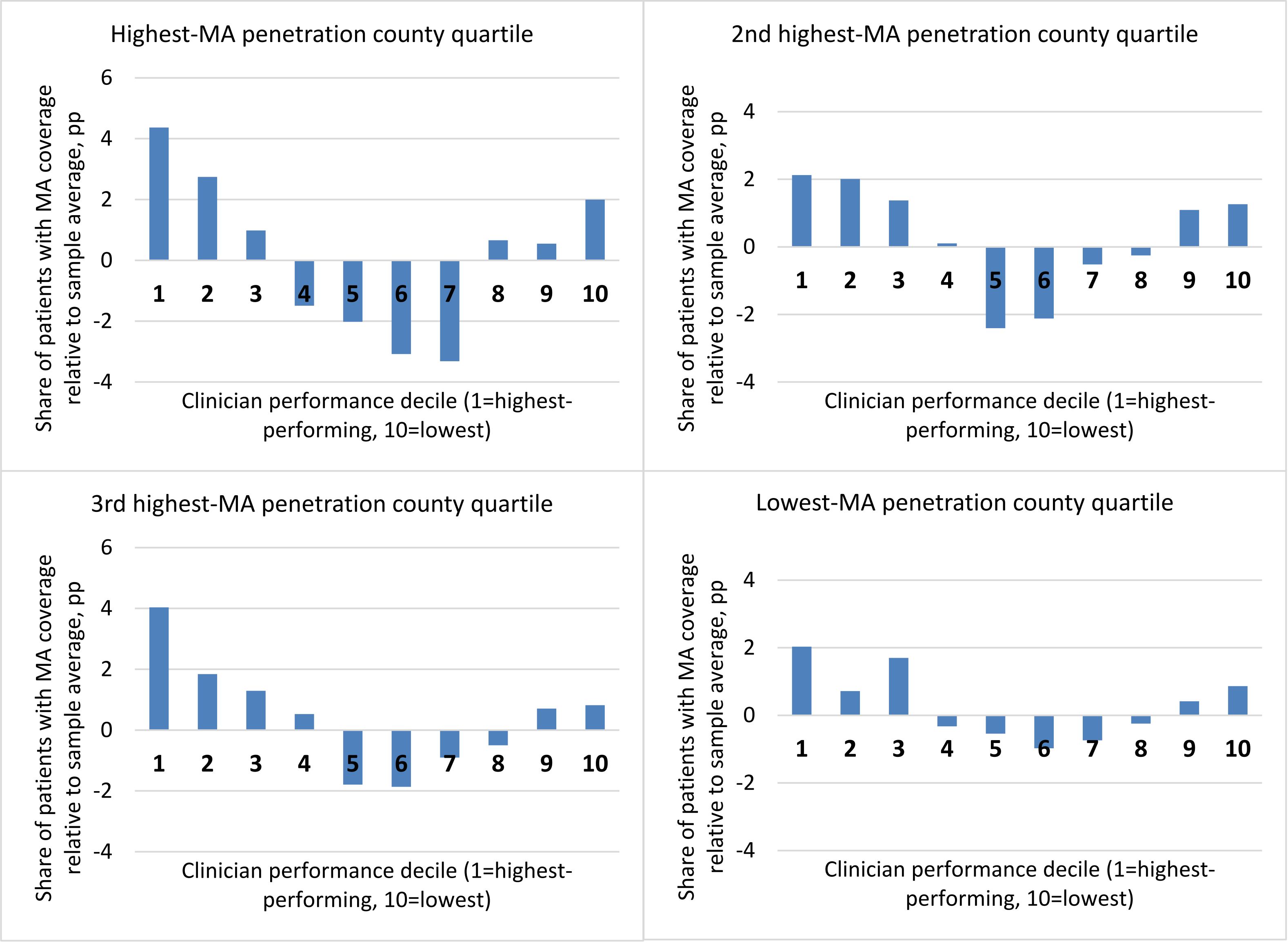

Note: This figure is based on the clinician fixed effect estimation sample, which excludes clinicians without any avoidable hospital stays among their patients. The performance deciles are based on avoidable hospitalizations among the clinicians' traditional Medicare patients during 2016-2018, with the first decile being the clinicians with the lowest adjusted rates. MA, Medicare Advantage.

© 2023 Xu JF et al. *JAMA Health Forum.*
