# Supplementary material for: Role of Patient Sorting in Avoidable Hospital Stays in Medicare Advantage vs Traditional Medicare
Source: JAMA Health Forum. 2023 Nov 10;4(11):e233931. doi: 10.1001/jamahealthforum.2023.3931 (PMC10638641; doi:10.1001/jamahealthforum.2023.3931)
Supplement: Supplement 2. — Data Sharing Statement [file jamahealthforum-e233931-s002.pdf]

# Data Sharing Statement

Xu. Role of Patient Sorting in Avoidable Hospital Stays in Medicare Advantage vs Traditional Medicare. *JAMA Health Forum*. Published November 10, 2023.
doi:10.1001/jamahealthforum.2023.3931

**Data**
**Data available:** No

**Additional Information**
**Explanation for why data not available:** We used restricted claims data obtained through a data use agreement with CMS, which prohibits data sharing with individuals outside the research team.
